# Breast cancer survivors suffering from lymphedema: What really do affect to corporeality/body image? A qualitative study

**DOI:** 10.1186/s13058-024-01806-9

**Published:** 2024-03-14

**Authors:** Laura González-Fernández, Carlos Romero-Morales, Beatriz Martínez-Pascual, Angela Río-González, Ester Cerezo-Téllez, Inmaculada López-Martín

**Affiliations:** 1 Faculty of Sport Sciences, Universidad Europea de Madrid, Villaviciosa de Odón, Madrid, Spain; 2 Neuromusculoskeletal Physical Therapy in Stages of Life Research Group (FINEMEV), Department of Nursing and Physical Therapy, Faculty of Medicine and Health Sciences, Universidad de Alcalá, Alcalá de Henares, Madrid, Spain; 3 Escuela de Enfermería Fundación Jiménez Díaz, Instituto de Investigación Sanitaria - Hospital Fundación Jiménez Díaz, Universidad Autónoma de Madrid (IIS-FJD, UAM), Madrid, Spain; 4 Asociación Española de Linfedema y Lipedema, AEL, Madrid, Spain

**Keywords:** Lymphoedema, Breast cancer, Physical therapy, Qualitative research, Distorted body image, Medical oncology, Phenomenology

## Abstract

Breast cancer-related lymphedema is currently one of the most serious complications that most affect the quality of life of women undergoing breast cancer. The aim of this study was to explore in-depth the experience of women who suffer from lymphoedema after breast cancer and how does this condition affect corporeality, with no judgements. For this purpose, a qualitative methodology was followed. In-depth interviews, interviewer's field notes and participants' letters were used for data collection. The participants were twenty Spanish women with lymphoedema after overcome a breast cancer in the past. Healthcare specialists with experience in the topic were also included. Results showed 2 main categories: “From cancer to lymphedema, another disease another disease” and “Potential for transition and transformation towards a new way of life”. As a conclusion, the difficulty in accessing adequate treatment, the need for greater awareness of lymphedema and the importance of the emotional and psychological dimension of this chronic disease. Highlighting the attitudes that these women develop for self-care and the concept of new corporeality. After breast cancer, women with lymphedema experience a drastic change that affects all areas of their lives. The adaptation process, and the search for resources and aid, play a fundamental role in overcoming this process.

## Introduction

Breast cancer is the most frequent tumor and the first cause of death in developed countries affecting about 3–5 million patients worldwide [29] with Spain being the second most affected country. Generally diagnosed between 35 and 65 years, approximately 1 of every 8 women will suffer a breast cancer once in their life. Even if the 89.2% of women in stade II, reaching a 98% in stade I [43] survive, thanks to medical advances in surgical or coadjutant proceedings, but mainly due to a prompt diagnosis screenings [3].

Breast cancer secondary lymphoedema, with an incidence between 2 and 70% (Tandra et al. [44] and Levenhagen et al. [21] is described as the most stressing complication after breast cancer in the long-term, not only for its psychologic problems (volume augmentation of the arm, non-recovery sensation, aesthetic distortion, decrease of self-esteem) but also it may produce functional handicap and other complications as infections (lymphangitis and erisipels) [46]), hyperkeratosis [24, 39, 41], fibrosis caused by the augmentation of collagen and inflammatory reactions [46] and pain axillary web syndrome [2]. It is currently one of the most serious complications affecting physical, social and emotional dimensions [4]. Furthermore, it reduces quality of life [4, 10, 31], sexuality and corporeality of breast cancer survivors [14, 16]. Several risk factors has been described for the development of a secondary lymphedema: body mass index (BMI), time to be diagnosed, subclinical edema and the appearance of cellulitis in the affected areas [28]. In this context, current evidence reported that regarding biomarkers to assess the biological effects of some interventions, such as physical exercise in a tailored rehabilitation program, could be benefit to their physical and psychological condition in these patients [17].

Psychological distress as anxiety and uncertainty are the worst feelings that cancer survivors support during their treatment [22]. Furthermore, sexual wellbeing is affected by hormonal and endocryne treatments disturbing quality of life [27]. In addition, the cognitive impairment [6, 34] insomnia and fatigue [34] added to the lack of sympathy and information of sanitary professionals [20] affect negatively the quality of life of these patients. The loss of roles and social dysfunction also provoques vulnerability [14], increasing every year after diagnosis [8]. Lymphoedema increases handicap, functional alterations corporeality, disease resiliency, job difficulties, family roles, lost of self-esteem and social isolation [12], provoking emotional responses as fear or frustration [40]. All these symptoms are more important if the lymphoedema appears in the dominant side developing higher difficulties on physical and psicological functioning with a lack of social support perception [12]. All these arguments make mandatory the need of psicologic and physical therapy strategies focused on prevention.

Corporeality as the self image every person has of his/her body, depends on individual (self-image, affective life or mood) and contextual (environmental opinions and fashion) circumstances [14]. It presents a high distress in breast cancer survivors.

Corporeality, quality of life and sexual function are also affected during and after breast cancer treatments [23]. Social pression over women population related to acquiring an aesthetic ideal, affects negatively over breast cancer survivors. It is due to bodily changes all over the disease process. This breakdown, related to the cultural-stereotype, conforms a psycologic stigmatisiting reflect because of surgery, with the extirpation of a part of the body, the breast, closely identified with feminity, self-esteem and sexuality [14, 16, 18]. In addition, changes in self-esteem and sexuality affect negatively in patients corporeality, being breast cancer diagnosis the harmfullest [38]

Other factors as age [19], type or surgical proceeding [42], reconstruction [13, 27], chemotherapy, radiotherapy, hormonotherapy [19, 30], sexuality [19] also affect.

The lack of research about corporeality is due to the difficulty of the conceptual or methodological quantification on the one hand the part of the sexuality on the other hand the part f self-image or self-concept [42].

Secondary lymphoedema after breast cancer contributes to the social pression towards body worship previously described with physiologic implication which also has corporeality repercussion [14]. Breast cancer secondary lymphoedema associated symptoms as range of movement limitation, rigidity, numbness, augmentation of volume impact negatively over recovery sensation, aesthetic distortion, decrease of self-esteem, disfiguration [12], function and physical wellbeing producing a quality of life decrease and other complex multidimensional effects over self-image, sexuality, social relationships and recreative activities [11].

This pathology, its chronicity [20, 39] and the lack of information about it [9, 24, 39], provoques a physical, phycosocial and emotional impact that affects the quality of life, economic stata, concerning money [31, 39], time and changes in the style of life [31, 40] and sexuality [37] of cancer survivors [39] Furthermore, sanitary personnel do not know about this disease [9, 31]. Breast cancer survivors feelings are uncertainty, disappointment, guilt and shame, security and autonomy [20] shock, fear, frustration and a negative self image [31]. In addition, to do an individual multidisciplinary approach is the clue [20].

The need of know-in-deep about how cancer survivors suffering from lymphoedema live this situation will help us to promote treatment strategies focused on the patient [20].

The aim of this study is to know the experiences, emotions, and feelings related to suffer lymphoedema after breast cancer and its repercussion on bodily image.

## Methods

### Design

This is a qualitative phenomenological study with descriptive perspective that aims to explore how cancer survivors experience suffering from lymphoedema. It was carried out in privates physical therapy healthcare centers of Madrid (Spain).

### Participants

The participants were 20 breast cancer survivors almost 1 year after their surgery, diagnosed with secondary lymphoedema homolateral to lymphadenectomy, over 18, with physical and psychological ability to understand and participate in the study, and understand and speak Spanish with. Recruitment was carried out through the physical therapy clinics. In addition, local announcements were posted in oncology care centers explaining the aim and procedures of the study. Exclusion criteria were women that did not sign informed consent, presence of any systemic disease, metastasis, neurological problems, and/or cognitive problems, primary lymphoedema or lymphoedema with a different etiology from described in inclusion criteria.

Participants were recruited until data saturation was reached, that is, to reach the moment in which new ideas or themes no longer appeared and the information obtained was redundant [25] when performing an exhaustive analysis of the data [32]. Finally, the sample consisted of 20 women, from whom age (range between 37 and 82 years, average age 56.15), marital status (14 married, 3 single, 2 divorced and 1 widow) was obtained as descriptive information, work activity carried out (14 work outside the home, 5 retired and 1 does not work outside the home), number of children (between 0 and 3, 1.22 children on average). We also collected the type of breast cancer surgery and the year it was performed, the type of treatment (chemotherapy/ radiotherapy/hormonal therapy), the time the lymphedema appeared, and the type of treatment applied, if any, in addition to whether received information on preventive measures and who provided them. The sociodemographic data collected help to understand the context and assess the transferability of the results obtained to other places and similar contexts.

They were recruited through the snowball method. Each participant was asked to sign the informed consent form prior to participate in the study.

### Ethical considerations

This study was approved by the Clinical Research Committee of Rey Juan Carlos university register number 03/2013. Before the study commenced, all participants signed an informed consent form. Ethical requirements for human experimentation and Helsinki Declaration were respected.

### Data collection

Data collection was carried out using four strategies: unstructured interviews, semi-structured interviews, interviewer's field notes, and personal letters with testimonials and stories from the participants.

The 12 unstructured interviews, linked to the type of sampling by purpose, allowed the participants to use their own language, without restrictions or rigid structure, to tell their story, their experiences, without interruptions from the researcher. For the 8 semi-structured interviews, corresponding to the theoretical sampling phase, open questions oriented to emerging topics of interest that required in-depth analysis were prepared. These questions were oriented towards experiences, behaviors, opinions or values, feelings and knowledge, sensory aspects and personal background [26].

All interviews were recorded and transcribed. Observations were also collected in the field notebook during its completion. A total of 20 anonymous interviews were carried out for treatment and analysis with the qualitative analysis program Atlas-ti 7.0. The average duration of the interviews was 36 min, in a range of 22–59 min.

Personal accounts and the researcher's field diary were used as complementary data sources. This narrative material written in the first person was considered of great value for its reflective content [45].

The researcher's diary was used continuously during the fieldwork and data analysis phase. Twenty field notes from the researcher were analyzed, which, as Pérez Serrano [36] refers, contemplate subjective interpretations and impressions, in order not to lose relevant information for later use throughout the analysis.

The quality criteria for the evaluation of qualitative studies proposed by Leininger [32] have been taken into account, guaranteeing the credibility and confirmation of the data, the interpretation of the meanings in their context and the search for patterns. Recurring of these, the deep analysis of the interviews and the possibility of transfer to other similar studies. Triangulation procedures were applied between primary sources, between different types of data sources and between researchers during the analysis [45].

The initial results were presented to a multidisciplinary panel of experts, independent of the research team, in order to verify the consistency of the results and verify the accuracy of the data [32, 33, 35].

Data analysis was carried out at the same time as data collection, following three levels of abstraction [7].

A first level in which fragments or segments of meaning that are grouped into descriptive categories are identified. These categories are structured into metacategories at a second level of analysis, taking into account the differences and similarities found between them. At the third level of analysis, the interpretive dimension of the metacategories is reached, relating them and identifying the main vectors or themes.

After the analysis, a thematic area named “Living with a new corporeality" was identified at the metacategory level, the results of which are presented in this work. It is made up of two categories, which emerge from 13 codes, coming from the grouping of 236 fragments of meaning.

## Results

One main meta category was identified based on the experiences of patients with breast cancer: “Living with a new body image” made up of two categories: (1) “Elements that characterize my new body image” and (2) “The impact of my new body image”.

### Elements that characterize my new body image

(6 cod y 165 US) The new body image of these women is marked mainly by mutilation of the breast (sometimes they cannot look at themselves in the mirror naked, some want reconstruction and others describe the fear of going through the operating room again), also by hair loss during chemotherapy and by the appearance of lymphedema.

To facilitate the monitoring and achievement of results, and their identification related, we provide some fragments extracted from participants' quotes:

I.2: I think that what has repercussions above all is at the family level, at the marital level, it is a very painful issue, I think it is very important to be psychologically well to be able to face it. It is very hard. It is very hard to see yourself in a mirror and see yourself mutilated… And even more so in the world we live in today, where physical appearance is so important, it is very hard, it is very hard to see yourself like this.

I.15: As I say to these: you don't know what it is. I have a boob… and I say: you don't know what it is.

I.15: From my own body and I did not dare because I was so tired of the treatment and once again going into the operating room, (…) I told them no, that I did not dare, that I was very tired from so much hospital and I say: and now we start again? I prefer to wear a prosthesis.

I.7: And what I've been through was fatal, of course, the hair, the hair for me the worst. I get up and see hair on the pillow, which later I had to shave completely, that's very difficult.

This distorted body image is conditioned because of the lymphedema appearance (sometimes during the oncological treatment or months or years later). It is used to be accompanied by sensations like discomfort, pressure, or pain. In most cases, when it appears, they do not know what is happening; this causes them fear of a possible recurrence of cancer or any unknown disease.

I.3: At first I didn't notice anything, the arm was great, everything was fine, but then I did begin to notice that it was more… and continues to be, more edematous. Above all, you notice it because your clothes are starting to not work for you, because they are starting to be too tight, it seems that they don't fit me.

I.17: And when it started to swell I was scared, yes I was scared.

The participants describe that the arm on the operated side is more swollen than their healthy arm, weighs more, causes fatigue, continuous discomfort, and limitations. Sometimes their volume increases with or without reason, or infections appear and worsen.

I.4: You feel like… you feel inflated like a balloon.

I.6: When it becomes very inflamed, hard and hot, the physical limitation is quite important.

I.1: But the arm kept growing, it didn't stop anymore, it was getting bigger, I couldn't close my hand, the fingers were little blood sausages, all day they gave me cream because the skin would have cracked, I slept with my arm up and I had a total obsession.

### The impact of my new body image

(7COD-71US) It is evident in the narrations the difficulty of accepting their new corporality after suffering the breast surgery, due to what symbolizes the breast in the femininity of a woman. Additionally, lymphedema also marks the new body image of these women, making the arm the center of attention of their daily life. Most participants point out the importance of their physical appearance. Both for themselves and their body image, and for others. No recognize their new body as their own. They feel asymmetrical both because of the chest and the lymphedema, and different. That makes them feel bad.

I.1: And then physically, your appearance, it's also hard to see that hand, the way I had it… and everyone asked me what was wrong with me… Of course, that deformed hand, well, yes, it affects… it affects… Me Most of all, my hand swelled up, more than other parts. Well, my side swelled, but since you couldn't see it, I didn't care. It mattered to me that people saw my hand.

I.6: Seeing the swollen arm does not attract attention (…) but when I see a photo of myself with the arm in close-up, it impresses me.

I.8: When I went to the psychologist of the Spanish Association against Cancer, I told her: “it's just that I'm not me anymore”. Of course, I am no longer me, I saw myself without a mother, before I was half what I am now.

I.16: Then you want your breast to be reconstructed because I am not at all comfortable with the arm, or with the breast, or anything. Anyway, I got fat, my arm got fat, they removed my chest, you look at yourself and you're not even symmetrical, it's a very strange thing.

I.11: Above all, it is the theme of always feeling, of always being there feeling different, special.

Mastectomized participants feel mutilated. They don't want to look in the mirror. These feelings sometimes affects them in their relationships with their environment, partner and sexuality.

I.2: It's very hard. It is very hard to see yourself in a mirror and see yourself mutilated… And even more so in the world we live in today, where physical appearance is so important, it is very hard, it is very hard to see yourself like this.

I.7: The kid saw me naked and said: “Mom and your breast?” and I wanted to die…

I.16: At first my husband didn't look at me directly, now I think he doesn't care more or less, he must have gotten used to the idea, but it bothers me. It is that he is going to touch my chest and I am already on my guard. Yes, it does affect me.

The arm affected by lymphedema becomes the center of their attention. Many times, they feel disabled and limited.

I.2: And it is a bit of a summary of my life: I go to bed with my arm and all day I hang on my arm a bit. It's a roll.

I.14: Four years have passed and the first thing in the morning I do is get up when I'm lying down and look at my hand and say: “Ugh! And he's still here, he hasn't left.

I.1: It is the same as a person who has only one arm. I have realized that this is the case, what happens is that your brain sends you contradictory orders. Because you have both, he sends you contradictory orders, but no, you have one. And you have to be aware that you only have one arm, because if you don't, you'll be harmed.

## Discussion

The psychological impact of breast cancer on participants in this study is mainly related to their physical appearance and new body image. They do not recongnize themselves when visualizing their asymmetrical body [1, 30]. Also report that physical sequelae entail psychological symptoms which are related to the adaptation to the disease, especially distorted Body image, diminished self-esteem, and asymmetry perception. All of these symptoms reduce health-related quality of life of breast cancer survivors.

This new-body-image-perception is mainly defined by their new breast, hair loss during chemotherapy treatment and lymphoedema. Our results are like those reported by Río-González et al. [40] in relation to psychological impact, mastectomy-associated trauma provokes bad feelings as the feel of amputation and changes in their body image, lymphoedema as well as the remaining scar and the memories associated with the process.

In addition, the breast as a symbol of femininity, makes even more difficult to assume their new corporeality. They feel different from other women, they feel mutilated, they are not able to look themselves naked in the mirror not to be seen naked by others. This has a great impact on their sexuality and, therefore, on their relationships.

Our results agree with those of Hoyle et al. [16] who describes that body image and sexual concerns are common, intense, and distressing for patients who have lymphedema secondary to breast cancer.

The results obtained by McClelland [27] match in highlighting the loss of breasts with loss of femininity, and in the case of breast reconstructions, with the strangeness on their own poorly perceived body-image. And they also agree with Rincón Fernández et al. [38], stating that in breast cancer the impact of the disease on the body image of patients stands out.

Focusing on the impact on femininity, Hashemi-Ghasemabadi et al. [15] add that a greater dissatisfaction with their body image after breast cancer treatment are those women who consider themselves very feminine and who value, their physical appearance and breast, as an important factor for their femininity and attractiveness.

Morales-Sánchez et al. [30] claim that the body image seems to be a predictor of psychological functioning and if they are satisfied with their appearance, they predict a better quality of life.

Hair loss during chemotherapy can become a traumatic factor for some women affecting their body image [19]. In addition, suffering from lymphedema increases the aesthetic defect. This fact not only affects their body image but also limits their daily living.

Ruber Martí et al. [41] agree with this approach stating that lymphoedema is considered as the most long-term stressing complication of breast surgery. Río-González et al. [40] affirm that lymphoedema is more distressing than cancer because those affected must learn to live with a chronic problem that requires daily attention which agrees with our results. They conclude that it is necessary to adapt their lives to a new situation.

In this sense, Gironés i Coromina et al. [14] states that the distorted body image produced by lymphedema is aggravated by social pressure, based on the cult of the body and the canon of beauty prevailing in society.

In addition, Maree and Beckmann [24] adds that the difficulty they have for dressing because of the arm volume, also negatively impacts their body image and femininity.

### Limitations and future lines

Due to the limitations caused by lymphedema, participants feel disabled. Fernández et al. [12], states that lymphedema considerably alters the quality of life of women due to an increase in disability, functional alterations and body image. Also Aymerich and Espallargues [5] states that it is a cause of disability. DeSnyder et al. [11] exposes how the symptoms of lymphedema negatively impact in physical and functional well-being, decreasing the quality of life with negative effects on body image, sexuality, social relationships and recreational and leisure activities. Based on the findings of the present study, some potential future lines could be developed, such as, interventional studies on lymphedema management, longitudinal studies on the psychological impact and emotional responses in patients who live with this condition focusing on resilience or adaptation strategies.

### Clinical applications

The present study helps to clinicians and researchers to improve the current knowledge about the corporeal and psychological impact of lymphoedema on women who have survived breast cancer. The present work highlighted the necessity for improved access to treatment, increased awareness of lymphedema and the importance of support for emotional well-being and self-care practices. These findings underscore the complex interplay between physical changes and their psychological ramifications and their impact on body image and mental health.

## Conclusions

Living with postmastectomy lymphedema is living with a chronic disease, differentiated from breast cancer, which manifests itself with limitations, loss of autonomy and changes in body image, which have a clear impact on their personal, family and professional spheres. Women see themselves with a different body, to which they have to adapt both in relation to their chest and in relation to their arms. While the new image of the arm due to lymphedema constitutes a stigma for these women, the loss of functionality acquires a meaning of disability, insufficiently recognized both socially and professionally. The adaptation process and the search for resources and aid, play a fundamental role in overcoming this process.

## Data Availability

Data and materials were available under formal request.
